# A framework for automatic heart sound analysis without segmentation

**DOI:** 10.1186/1475-925X-10-13

**Published:** 2011-02-09

**Authors:** Sumeth Yuenyong, Akinori Nishihara, Waree Kongprawechnon, Kanokvate Tungpimolrut

**Affiliations:** 1 Department of Communication and Integrated Systems, Tokyo Institute of Technology, Japan 2-12-1-W9-108 Ookayama, Meguro-ku, Tokyo, 152-8552 Japan; 2 Department of Information, Computer and Communication Technology, Sirindhorn International Institute of Technology (SIIT), Thammasat University, Thailand 131 Moo 5, Tiwanont Road, Bangkadi, Muang, Pathum Thani 12000, Thailand; 3 Industrial Control and Automation Laboratory, National Electronic and Computer Technology Center (NECTEC), Thailand 112 Paholyothin Rd., Khlong Neung, Khlong Luang, Pathum Thani 12120, Thailand

## Abstract

**Background:**

A new framework for heart sound analysis is proposed. One of the most difficult processes in heart sound analysis is segmentation, due to interference form murmurs.

**Method:**

Equal number of cardiac cycles were extracted from heart sounds with different heart rates using information from envelopes of autocorrelation functions without the need to label individual fundamental heart sounds (FHS). The complete method consists of envelope detection, calculation of cardiac cycle lengths using auto-correlation of envelope signals, features extraction using discrete wavelet transform, principal component analysis, and classification using neural network bagging predictors.

**Result:**

The proposed method was tested on a set of heart sounds obtained from several on-line databases and recorded with an electronic stethoscope. Geometric mean was used as performance index. Average classification performance using ten-fold cross-validation was 0.92 for noise free case, 0.90 under white noise with 10 dB signal-to-noise ratio (SNR), and 0.90 under impulse noise up to 0.3 s duration.

**Conclusion:**

The proposed method showed promising results and high noise robustness to a wide range of heart sounds. However, more tests are needed to address any bias that may have been introduced by different sources of heart sounds in the current training set, and to concretely validate the method. Further work include building a new training set recorded from actual patients, then further evaluate the method based on this new training set.

## 1 Background

Heart disease is a major health problem and a leading cause of fatality throughout the world. Treatment can be easier and cheaper if the condition is detected early. Cardiac disorders that are valve related can be detected efficiently and cheaply using *auscultation*. Unfortunately, auscultation requires extensive training and experience to perform effectively, and such training has been on the decline due to the availability of new cardiac examination technologies [1]. Regardless, auscultation remains the most cost-effective method for cardiac examination because it requires minimal equipment. This makes auscultation the primary and often the only means of cardiac examination available for small primary health care clinics. In reality, however, medical personnel in these clinics have little or no training in auscultation. The benefits of automatic auscultation can be considerable.

Auscultation consists of two phases: heart sound acquisition and heart sound analysis. Heart sound acquisition involves placing a stethoscope at the appropriate location on a patient's chest with the right amount of force to capture heart sound. Heart sound analysis is used to determine whether or not the captured sound corresponds to a healthy or diseased heart. This work is part of a larger study whose aim is to create a remote auscultation system, a robotic device that can perform both heart sound acquisition and analysis. Since acquisition is mostly a mechanical process, this work focuses only on the analysis. The work focuses further on only detecting the presence of a disorder, but does not attempt to identify it. That is, heart sounds are classified into two classes: "healthy" or "diseased". While this is a simpler problem than the multi-class problems (providing diagnosis of the type of the disease) addressed by most studies on heart sound analysis, it allows for a method that does not require heart sound segmentation and thus, is robust to sounds that are difficult to segment due to significant signal corruption.

### 1.1 Heart Sounds and Cardiac Cycles

**Figure 1:**
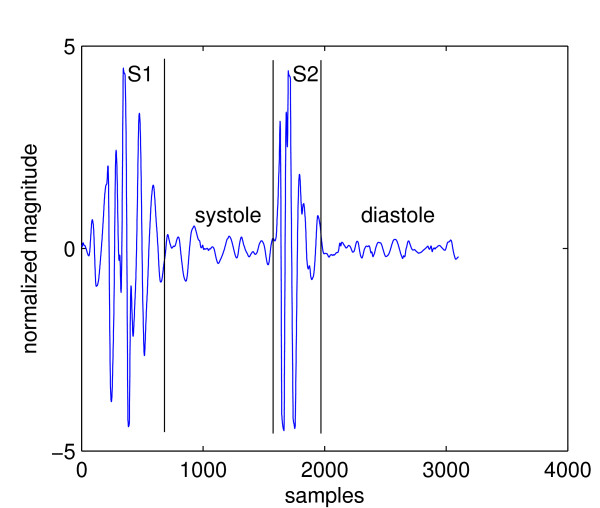
**A normal cardiac cycle**.

Heart sounds in healthy adults consist of two events: the first heart sound (S1) and the second heart sound (S2). Together they are referred to as *fundamental heart sound *(FHS). A cardiac cycle or a single heartbeat is defined as the interval between the beginning of S1 to the beginning of the next S1. The interval between the end of S1 to the beginning of the same cycle's S2 is called *systole *and the interval between the end of S2 to the beginning of the next cycle's S1 is called *diastole*. In, general systole is shorter than diastole, which is the assumption that most heart sound segmentation methods are based on. Figure 1 shows a cardiac cycle with the components separated by black vertical lines.

### 1.2 Abnormal Heart Sounds

**Figure 2:**
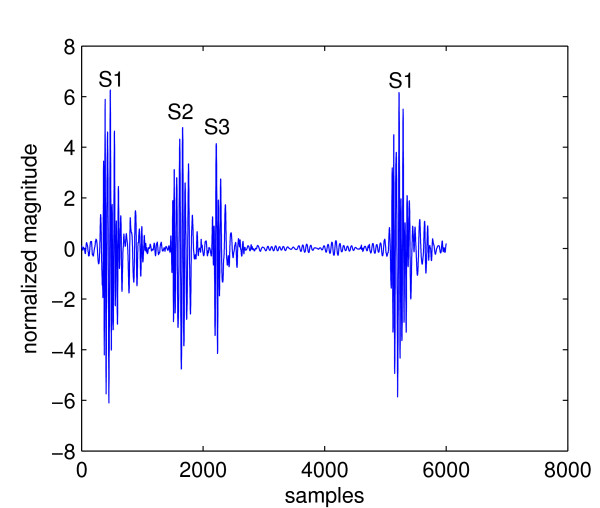
**A cardiac cycle with third heart sound**.

**Figure 3:**
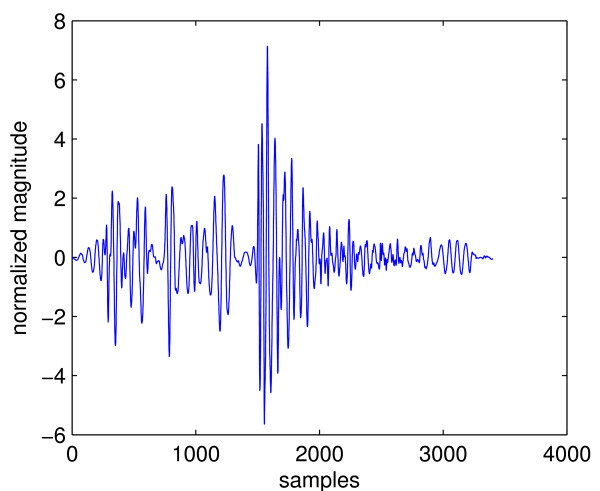
**A cardiac cycle with aortic regurgitation**.

Cardiac cycles of abnormal heart sounds contain components that are not one of the FHS. These components can be grouped into two types: extra heart sounds and murmur sounds. An example of the first type is the third heart sound (S3) shown in Figure 2. The second type of abnormal heart sound is called a murmur. They are caused by turbulence blood flow through a blocked (stenosis) valve, or backward flow through a leaking (regurgitation) valve. These sounds can be heard both in systole or diastole, depending on the underlying condition. The presence of murmur is a good indicator of valvular (valve related) disorders. Figure 3 shows a cardiac cycle with a murmur called aortic regurgitation caused by leakage of the aortic valve. It can be seen by comparing Figure 2 and 3 that murmurs are fundamentally different from normal heart sounds. Whereas S3 looks like an extra copy of FHS, murmurs alter the waveform of a cardiac cycle completely and the locations of FHS can no longer be marked precisely. This can cause problems for heart sound analysis in the segmentation state because most segmentation algorithms are based on determining the locations and types of FHS. It can be seen that abnormal heart sounds are characterized by the presence of extra components in their cardiac cycles that are not FHS. Thus one could formulate a heart sound analysis problem as detection of components other than FHS in cardiac cycles.

### 1.3 Overview of Heart Sound Analysis

Heart sound analysis can be broken into three generic steps: segmentation, feature extraction, and classification. Segmentation is to determine the boundaries of cardiac cycles from contiguous heart sound signals. Feature extraction is to calculate the identifying parameters from each cardiac cycle. classification is to make a decision on a heart sound's type based on those parameters.

There are two approaches to segmentation: envelope based and machine learning based. Most existing algorithms use the envelope approach since it is not necessary to label different components of cardiac cycles manually. On the other hand, if labelled cardiac cycles are available, then a machine learning approach is preferred because tedious envelope analysis can be avoided. The details of segmentation is discussed next.

### 1.4 Segmentation

Segmentation has been the subject of many studies because it is the first and perhaps the most difficult step in heart sound analysis. The most popular approach to segmentation can be called the "envelope analysis" approach. This approach calculates the envelope signal of a heart sound, detects peaks of the envelope signal, establishes which peaks correspond to S1 and which correspond to S2 and, then forms cardiac cycles using the S1-S1 intervals. Examples of this approach include [2], using average normalized Shannon energy, [3] using homomorphic filtering, [4] using complexity signatures, [5] using energy of wavelet coefficients and [6] comparing various envelope detection methods. All these studies fall under the envelope analysis approach since they are all based on analysis of the envelope signals and are different only by envelope detection methods. Due to its widespread use, the envelope analysis approach in general is discussed next.

#### 1.4.1 Segmentation of Normal Heart Sounds

**Figure 4:**
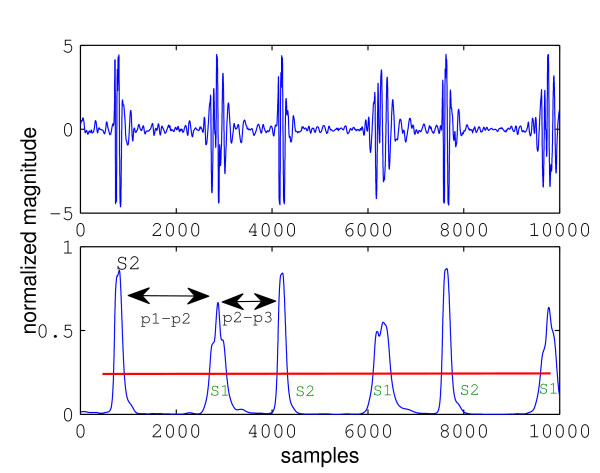
**Segmentation of normal heart sound**.

Figure 4 shows a plot of healthy heart sound and its envelope signal calculated using the homomorphic filtering method. Peaks in the envelope signal correspond to the FHS, which in healthy heart sound can all be easily detected by thresholding. The threshold is indicated by the horizontal line. A peak is defined as a segment of signal between two consecutive threshold crossings and its location is marked at the maximum sample in the segment. Any group of three consecutive peaks is then analysed. The distance between the first and second peak (p1-p2 in Figure 4) is calculated and compared with the distance between the second and third peak (p2-p3). Based on the assumption that systole is shorter than diastole, it is easy to identify systole as the shorter interval; thus, S1 must be the peak to the left of systole, or equivalently, S2 must be to the right. The identify of just one peak allows all other peaks to be identified by noting that S1 and S2 must alternate. Boundaries of cardiac cycles are then formed by the S1-S1 intervals and segmentation is completed.

#### 1.4.2 Segmentation of Abnormal Heart Sounds

**Figure 5:**
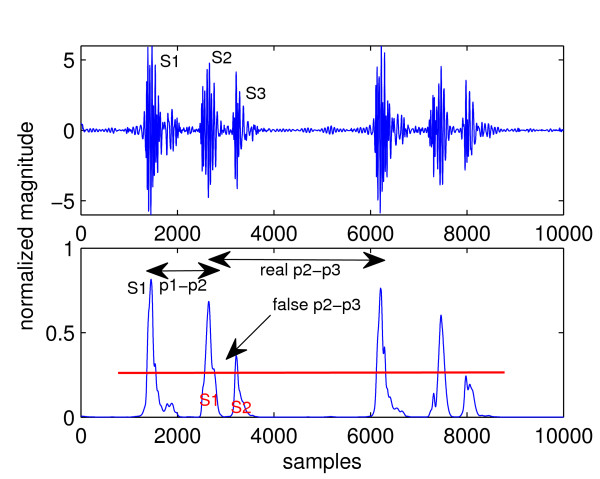
**Segmentation of heart sound with S3**. Note the error caused by the third peak.

This section illustrates problems that arise when an abnormal sound is segmented. Figure 5 shows the envelope signal of a heart sound with S3. Suppose that the three leftmost peaks are analysed, which actually correspond to S1, S2, and S3, respectively. The third peak is an *extra *peak such that it does not correspond to any FHS. Due to the presence of this peak (marked as "extra peak" in Figure 5), the length of p2-p3 interval has changed. It is no longer the distance between the second and the fourth peak, like it should be without the extra third peak, but the distance between the second and third peak. This is shorter than the p1-p2 interval and leads to the false conclusion that the second peak is S1. Using this peak as a reference to label all other peaks leads to wrong identification of all peaks in the envelope signal and ultimately wrong segmentation results.

**Figure 6:**
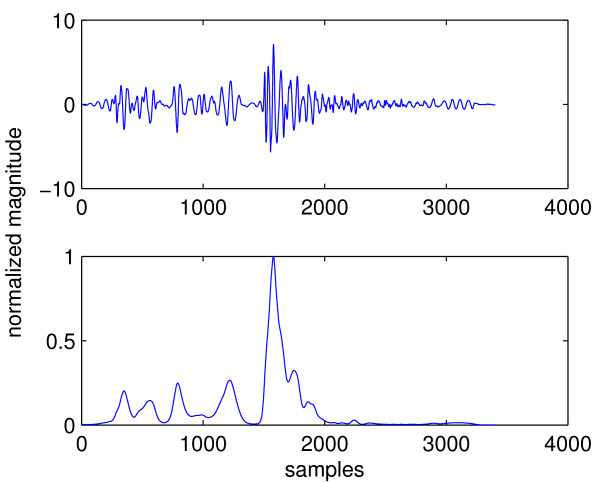
**A cardiac cycle with aortic regurgitation and its envelope signal**.

Segmentation is even more difficult in the case of heart sound with murmur. Figure 6 shows a cardiac cycle with aortic regurgitation murmur and its envelope signal. Aortic regurgitation is characterized by diastolic murmur, in addition to diminished S1 sound [7]. From the figure it can be seen that just one cardiac cycle contains 4 peaks, the largest of which corresponds to S2. However, there is no clear location of S1 as can be seen from the top panel, where S1 appears to have been "squashed". Therefore the envelope signal has 3 peaks that can not be labelled because the location of S1 is uncertain. For this reason, segmentation using the envelope analysis approach needs to eliminate extra peaks while retaining the ones that correspond to FHS. This is called "peak conditioning". In general, peak conditioning is based on "minimum peak interval". That is, if an interval between consecutive peaks fall below the minimum interval, it indicates that one of them must be an extra peak; and a common procedure is to eliminate the one with smaller magnitude. Peak conditioning can become a very tedious process due to several reasons:

1. Depending on where and how hard the stethoscope is placed on the chest, the FHS peaks may actually be smaller in magnitude than the extra peaks. Thus a threshold could be too high, so that one or more FHS are missed. This means that the missing FHS peaks must also be detected; if this is done by lowering the threshold, more extra peaks could be detected.

2. In cases with severe murmur, one of the FHS may become very large and the other may disappear from the envelope signal altogether.

3. The assumption that systole is shorter than diastole is not always true. The length of diastole decreases with increasing heart rate; above a certain heart rate it becomes roughly the same length as systole.

4. Some cardiac cycles may be incorrectly segmented and there is no way to automatically distinguish correctly segmented cycles from incorrectly segmented ones. Allowing incorrectly segmented cycles to enter feature extraction results in bad training vectors for the classifier.

Due to difficulties in the envelope analysis approach, segmentation methods based on machine learning such as hidden Markov models (HMM) or time-delayed neural network (TDNN) have been proposed [8,9]. Using these approaches, tedious envelope analysis can be avoided at a cost of having to prepare training data for the HMM or TDNN by manual segmentation. That is, a training set has to be hand-labelled by a human expert. In practice however, cardiologists often use the carotid pulse which can be felt in the neck artery during systole to aid in identifying the FHS. Therefore labelling a heart sound by visual inspection of the waveform alone can be challenging even for an expert. It can be seen that segmentation is a difficult problem, but under the method proposed in this work, it is not necessary to identify the FHS. The only information needed is the auto-correlation of the envelope signal, which makes it applicable even for heart sound waveforms that have been heavily corrupted by extra components.

### 1.5 Feature Selection and Extraction

Feature sets found in the literature can roughly be grouped into two types. The first employs medical knowledge about specific diseases and how they affect the generation of heart sounds. An example of a feature of this type is the split S2 interval. Many cardiac disorders cause S2 to split into two separate sounds. Other types of features are based on time-frequency signal representations. This type of representation is particularly suitable for heart sounds since they are non-stationary signals whose frequency content changes with time. A particular time-frequency representation commonly used in heart sound analysis is the discrete wavelet transform (DWT) [10]. Other types of time-frequency representations of heart sounds were studied in [11] and it was shown that DWT is the most suitable representation. DWT had been used repeatedly for features in heart sound analysis by dividing detail level 2 (d2) coefficients into non-overlapping portions, then, calculating the signal power of each to form elements of a feature vector [12-14]. DWT based features offer two advantages over the first approach. They are well-studied in signal processing and many software packages exist for their calculation. This makes them easier to implement, compared to ad-hoc features extraction. Moreover, DWT coefficients are unaffected by the type of envelope detection method used, since they are calculated directly from heart sound signals.

### 1.6 Using the DWT for Heart Sound Segmentation

**Figure 7:**
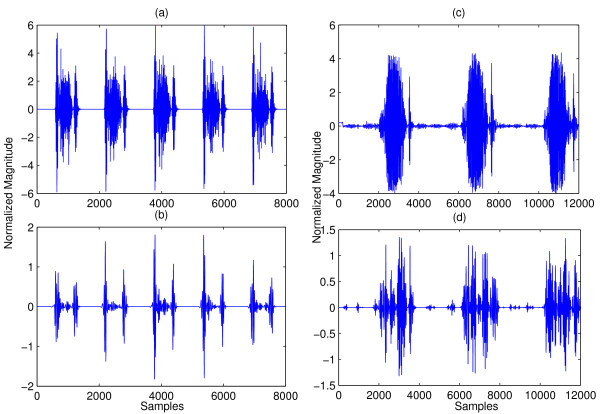
**Comparison of DWT denoising on heart sounds with different murmur intensity. **(a) Heart sound with relatively mild aortic stenosis. (b) Reconstructed signal from d5 DWT coefficients. The murmur had been attenuated significantly. (c) Heart sound with severe aortic stenosis. (d) Reconstructed signal from d5 DWT coefficients. Murmur is still largely present.

The DWT had also been used for heart sound segmentation. This method is based on decomposing the heart sound to be segmented and then reconstruct the signal using using only some of the DWT coefficients such that murmur is removed from the reconstructed signal. For example in [15,16], the heart sound signal was decomposed using db6 wavelet with 5 levels then reconstructed using the a4, d5, d4 and d3 coefficients separately or some of their combinations. The reconstructed signal that had the most murmur removed went on to envelope detection. The envelope calculated from this signal clearly shows the location of the FHS, since murmur has been removed, which allows for easy segmentation. Thus, by reconstructing the signal using only a certain coefficient levels, murmur can be separated from FHS because they do not overlap in frequency bands that correspond to the DWT coefficients used to reconstruct the signal. However in this study it was found that this may not always be the case because:

1. Often murmur can be eliminated by DWT decomposition and reconstruction. However, when a murmur is very loud, it overlaps with the FHS both in time and in frequency, and hence cannot be eliminated by the DWT method. Figure 7 compares the result of applying the DWT method described in [15] on a heart sound with moderate murmur and that with a very loud murmur. It can be seen in the left column that the murmur has been significantly removed. On the other hand, in the right column, the murmur is still largely present.

2. The extra heart sounds S3 and S4 are very similar to S1 and S2 in terms of frequency content. Therefore the DWT method cannot eliminate these sounds and their presence could compromise subsequence segmentation.

3. Some types of cardiac disorders can make S1 or S2 disappear altogether, such as severe aortic stenosis. Any segmentation algorithm based on locating the FHS would not work in this case.

For these reasons, it can be seen that DWT may not always work for heart sound segmentation in general. In fact, in the literature, there is no segmentation algorithm which claims to work on all types and degree of severity of abnormal heart sounds. Thus in this work we proposed a method that does not require segmentation altogether.

## 2 Method

**Figure 8:**
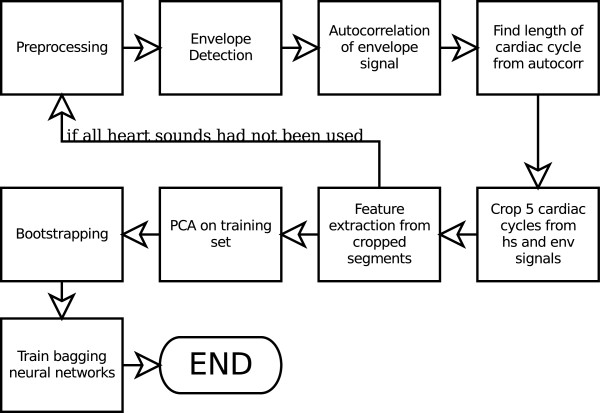
**Proposed heart sound analysis method**.

The heart sound analysis framework proposed in this work is shown in Figure 8. The main advantage of this approach is that it allows analysis to proceed without having to label each FHS. This is a big benefit for heart sounds with severe murmur whose FHSs are so corrupted that it becomes impossible to segment such sound. Obviously, such sound is abnormal, but in a traditional heart sound analysis approach that requires segmentation, it cannot be analysed, or could be miss-classified because of incorrect segmentation.

### 2.1 Preprocessing and Envelope Detection

Preprocessing consists of down-sampling and noise-removal of raw heart sound signals. In this work a sampling frequency of 4000 Hz was used. Each sound in the training set was either down-sampled or interpolated and re-sampled depending on its original sampling frequency. De-noising is based on DWT thresholding [17], with decomposition and thresholding parameters the same as that in [18].

Envelope signals were obtained from pre-processed heart sound signals by the following equations, where *x *denotes heart sound signals and *E *denotes envelope signals [5]:

(1) $$\psi_{m}(k)=\pi^{-1/4}\exp(\frac{-k^{2}}{2a_{m}^{2}})\exp(j\omega_{m}k)$$

(2) $$Y(m,\;n)=\sum_{k=1}^{N}\psi_{m}(k-n)x(k)$$

(3) $$E(n)=\sum_{m=1}^{M}|Y(m,\;n){|}^{2},\;m=1,2,\ldots ,N.,$$

where *ψ*_*m*_(*k*) defines the mother wavelet of a Complex Morlet wavelet with scale parameter *a_m_*. Equation 2 defines a *scalogram*, a 2-dimensional representation of a signal where one variable is time and another is scale. *Y *is a matrix whose rows correspond to scale and whose columns correspond to time. Equation 3 takes the magnitude of each element in *Y *and sums each column to get the envelope signal *E*. The scale parameters *a_m _*were chosen such that they correspond to a frequency range of 10 to 300 Hz, where the frequency bins are logarithmically spaced with 8 bins per octave. This means that the frequency range between 10 and 20 Hz was divided into 8 bins, and the frequency range between 20 and 40 Hz was also divided into 8 bins. This repeats until 300 Hz. The corresponding scales to these frequency bins were obtained by:

(4) $$s=\frac{F_{c}}{F\Delta },$$

where *F *is the frequency, *F_c _*is the center frequency of the mother wavelet, which is 5 rad/s for Complex Morlet wavelet, and Δ is the sampling period. This approach to envelope detection has an advantage over other approaches such as those in [2,3]. Since the shape of Complex Morlet is very similar to that of an FHS, *E *will have high peaks where FHS occur and will be attenuated everywhere else.

### 2.2 Determine Lengths of Cardiac Cycles

**Figure 9:**
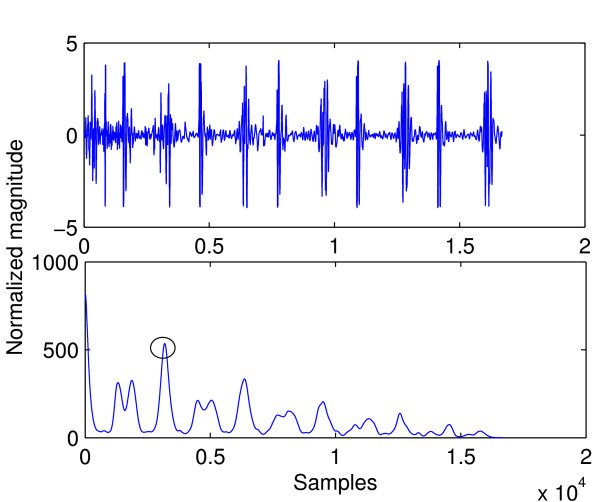
**A heart sound signal and the auto-correlation of its envelope**.

Envelope signals were used to determine lengths of cardiac cycles by employing their autocorrelation [8,19],

(5) $$R_{xx}(m)=\{\begin{array}{ll} \sum_{n=0}^{N-m-1}x(n+m)x(n) & m\geq 0,\\ R_{xx}(-m) & m<0 \end{array}$$

where *N *is the signal length. Only positive values of m need to be considered since heart sound signals are real. The length of a cardiac cycle was determined by seeking for the first peak after the origin in the envelope of its autocorrelation function. Since heart sounds are nearly periodic signals, auto-correlation of envelope signals will have peaks where the shifted signal *x*(*n *+ *m*) has been shifted by exactly one period and its cardiac cycles are aligned with those of the unshifted *x*(*n*). Figure 9 shows a heart sound signal in the top panel and the auto-correlation of its envelope signal in the bottom panel. The waveform in the bottom panel was searched for a maximum within the domain of 1000 to 5000 samples from the beginning of the signal. At *f_s _*= 4000 Hz this domain corresponds to heart rates of 48 to 240 beats per minute (BPM), which account for usual human heart rates. The BPM information also allows for identification of abnormal heart rates. The peak marked by a circle is the maximum sample. It can be seen that the distance (number of samples) from this sample to the start of the signal is roughly the same as the length of the first cardiac cycle in the top panel. The assumption is that heart rate remains constant throughout the entire signal.

The lengths of cardiac cycles were used to crop 5 cardiac cycles worth of signal from both heart sounds and their envelopes. Cardiac cycle lengths allow for extraction of the same amount of information from heart sounds with different heart rates. This is necessary because during feature extraction some feature values are dependent on the number of cardiac cycles extracted. Henceforth "heart sound/envelope segment" refers to a segment of either heart sound or envelope with lengths equal to five cardiac cycles. The choice of 5 cycles is to strike a balance between the nice averaging effect of analysing multiple cycles, such that any local abnormality in any one cycle (impulse noise for example) will not have much effect on feature vectors, and the amount of processing required. There is also a practical reason: short auscultation listening time which decreases the time it takes to examine a patient.

### 2.3 Feature Extraction

**Figure 10:**
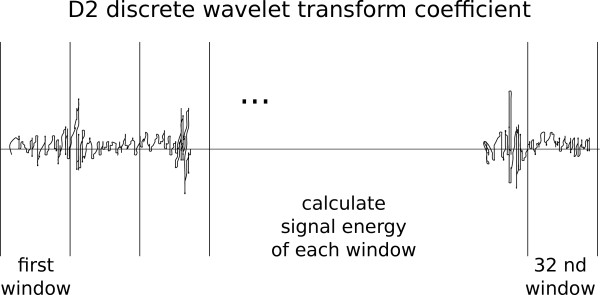
**Feature extraction from heart sound segment**.

**Figure 11:**
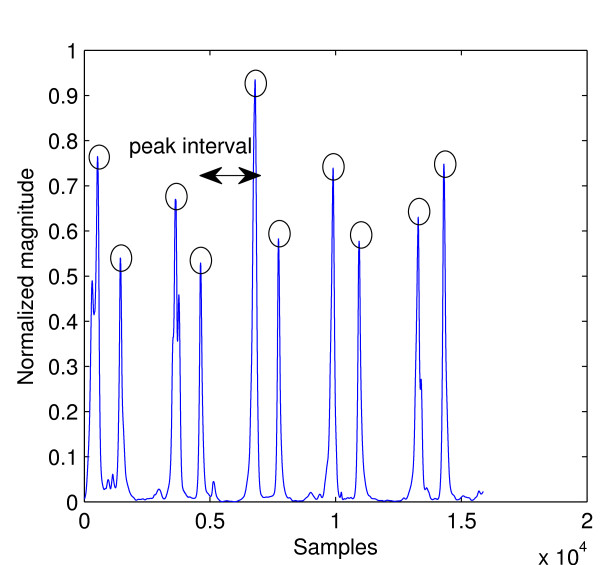
Envelope signal with 5 cardiac cycles.

The feature set used in this work consists of two parts: three features come from the envelope segments and another 32 come from heart sound segments. Features were obtained from heart sound segments using the same method as in [12] where d2 DWT coefficients decomposed for six levels using Daubechies-2 wavelet were partitioned into 32 non-overlapping windows and the signal energy of each window was used as a feature. This procedure is illustrated in Figure 10. It yields a 32-element feature vector for each heart sound segment. Another three features were obtained from envelope segments. Figure 11 shows an envelope segment. Each peak is marked with a circle and a peak interval and is shown by the arrows. The features derived from it were: the number of peaks, the average distance (in samples) between consecutive peaks, and the signal energy of the whole segment. The peak detection algorithm used to obtain the first two of these features is an improvement over simple thresholding; its pseudo code is shown below.

**M **= blank 1 by 2 matrix

*x *= 1, 2, ..., length(*E*) {*E *is envelope signal}

*m_x _*= -∞

*mxpos *= 0

**for ***i *= 1; length(*E*) **do**

   *t *= 1

   **if ***t *>*m_x _***then**

      *m_x _*= *t*

      *m_xpos _*= *x*(*i*)

   **end if**

   **if ***t *<*m_x _*- Δ **then**

      $\text{M}=\left[\begin{array}{cc} \text{M} & \\ m_{xpos} & x \end{array}\right]$ {concatenate the row vector $\left[\begin{array}{cc} m_{xpos} & x \end{array}\right]$ vertically with matrix **M**}

   **end if**

end for

**Figure 12:**
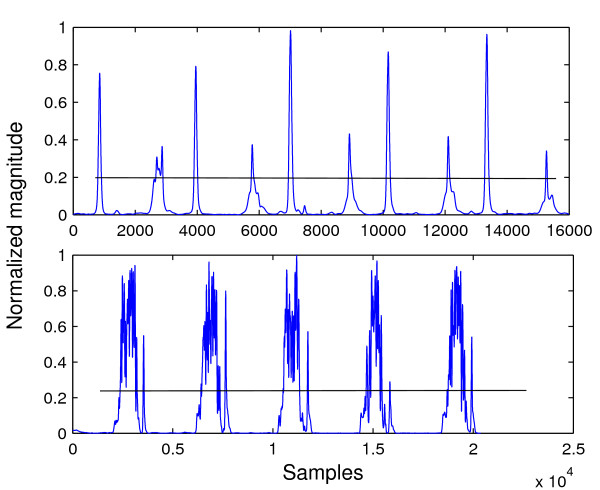
**Comparison between envelope segment of a normal heart sound and one with murmur.** (top) Envelope segment of normal heart sound. (bottom) Envelope segment with aortic stenosis murmur.

The difference between the above peak detection algorithm and simple thresholding can be seen by considering Figure 12 which shows a heart sound segment with severe aortic stenosis. If the threshold was set at around 0.25 as indicated by the black lines, simple thresholding would detect the same number of peaks as the number of threshold crossings divided by two; since there are many peaks above the threshold level, the result would be wrong. However, the peak detection algorithm used in this work would detect the correct number of peaks because in this case a peak is considered to be a maxima whose value is higher than the two "valleys" on either sides, by a threshold. That is, the threshold is compared from the top of a maxima, not from the x-axis. Finally for the third feature, the sum of all samples in a segment were simply added. The 3-element vectors and 32-element vectors were concatenated, forming 35-element feature vectors.

### 2.4 Principal Component Analysis

Principal Component Analysis (PCA) is a linear transformation method. In this work the following form was used:

(6) A=QB,

where **A **and **B **are matrices whose columns are feature vectors and **Q **is a matrix whose rows are eigenvectors of the covariance matrix of the training set **C**, arranged in decreasing order of magnitude of their eigenvalues from the top to bottom row. **C **is defined as:

(7) $$C=\frac{1}{n-1}\sum_{i=1}^{n}\left({\text{a}}_{i}-\bar{a}\right){\left({\text{a}}_{i}-\bar{a}\right)}^{T},$$

where *n *is the number of feature vectors, **a***_i _*are the raw feature vectors, and **ā **is the mean vector. The use of PCA allows for a significant dimension reduction for the feature vectors. This is because only eigenvectors whose sums of eigenvalues make up 90% of the sum of all eigenvalues are included in **Q **(the ones with larger eigenvalues were included first); and **C **usually has many eigenvectors with small eigenvalues and few with large ones. The next pseudo code demonstrates the use of PCA on a raw feature vector set, where the vectors are arranged as columns of matrix **X**.

**C**= 38 by 38 matrix of zeros

*N *= number of columns of **X**

**for ***i *= 1 → *N ***do**

   $\text{C}=\text{C}+\left({\text{x}}_{\text{i}}-\bar{x}\right){\left({\text{x}}_{\text{i}}-\bar{x}\right)}^{T}$

end for

$\text{C}=\frac{\text{C}}{N-1}$

calculate eigenvalues and eigenvectors of *C*

Let $E=[v_{1}^{T},\ldots ,\;v_{N}^{T}]$ where each row of *E *is an eigenvector and

Let *D *= [*d*_1_, ..., *d_N_*] their eigenvalues, where *d_i _*≥ *d*_*i*+1_

*S *= sum of *D*

*k *= *Q *= 0

**while ***Q *≤ 0.9*S ***do**

   *Q *= *Q *+ *D*(*k*)

   *k *= *k *+ 1

end while

*E *= *E*(*k *+ 1, ..., *N*) (drop the *k *+ 1^th ^till *N*^th ^rows of *E*)

**for ***i *= 1 → *N ***do**

   **y_i _**= **Ex_i _**(Each **y_i _**is a new feature vector)

end for

### 2.5 Bootstrapping and Bagging Classifiers

Let the original set of feature vectors and their class labels be denoted as *T *= {**x***_i_*, *d_i_*}, were **x***_i _*is a feature vector and *d_i _*its class label. After PCA, *T *was transformed into $T^{\prime }=\{{{\text{x}}^{\prime }}_{i},d_{i}\}$, where ${{\text{x}}^{\prime }}_{i}$ denotes feature vectors that have been transformed by PCA. Bootstrapping is to randomly sample from *T*' to generate *N *new training sets ${T^{\prime }}_{1},\;{T^{\prime }}_{2},\ldots ,{T^{\prime }}_{N}$, such that each is the same size as *T *. The sets ${T^{\prime }}_{i}$ were training sets for *N *individual neural networks (from here referred to as a network) which were trained independently using their respective training sets. During training, some members from each of the ${T^{\prime }}_{i}$ were reserved for testing, that is, were excluded from training. Each network was trained, then, its performance was evaluated on the reserved test set. Then it was retrained and evaluated again for 30 times. The network instance that had the best performance was kept. This process was repeated *N *times for all of the networks. Once all networks were trained they form "bagging classifiers", which are multiple classifiers working together to classify an input. An unknown feature vector can be classified by taking the vote of the outputs of the networks by:

(8) $$\forall i\in L,n_{i}=\{\begin{array}{ll} 1 & \text{if}\;Y_{i}\geq T\\ 0 & \text{otherwise} \end{array}$$

(9) $$c=\{\begin{array}{ll} 1 & \text{if}\;\sum_{i=1}^{N}n_{i}\geq \text{floor}\left(\frac{N}{2}\right)\\ 0 & \text{otherwise} \end{array}$$

where *L *is a set of all networks, *Y_i _*the output of a network *i*, *n_i _*an intermediate variable that counts the number of networks whose output is greater than or equal to threshold *T *, *N *is the number of networks and *c *is the class label. It has been shown in [20] that the classification performance of bagging predictors is always equal to or greater than that of a single classifier. Therefore, unless limited by computational resources, bagging classifiers are preferred over a single classifier. An unknown heart sound to be classified would pass through all steps in Figure 8, up to and including PCA, using the transformation matrix **Q **derived from the original training set *T*. The output of which becomes the input of all the *N *neural networks, and the decision of the heart sound type is made according to Equation 8 and 9.

In summary, the proposed heart sound analysis framework consists of:

1. Preprocessing that consists of down-sampling and noise-removal.

2. Envelope detection using Equation 1-3.

3. Calculate auto-correlation function of envelope signals.

4. Detect the location of the first peak in the domain of 1000 - 5000 samples after the origin of auto-correlation functions to find lengths of cardiac cycles.

5. Crop segments of five cardiac cycles from the heart sounds and the envelope signals.

6. Feature extraction from heart sound segments using DWT and from envelope segments using the peak detection algorithm in Section 2.3 to form 35-element feature vectors.

7. Perform PCA on the feature vector set *T *using the second algorithm in Section 2.3.

8. Perform bootstrapping on *T' *to generate *N *training sets.

9. Train each network with its training set.

10. The trained networks classify heart sounds using Equation 8 and 9.

## 3 Results and Discussion

The method proposed in this work was tested on a set of 57 individual heart sounds. Some of these sounds were recorded with a Thinklabs Rhythm ds32a electronic stethoscope, while others were obtained from several online databases [21-24]. Table 1 lists the types of sounds where the (m) indicates murmur. Two main experiments were carried out. The first was conducted to determine the optimal parameter set for bagging classifiers. These parameters are the number of hidden neurons of each network, and the decision threshold above which a network's output is considered positive (abnormal sound). The second experiment was conducted using the optimal parameters obtained from the first experiment to assess the classification performance, the robustness under white noise, random-value impulse noise, and unseen abnormal sound type of the proposed method. Since the main advantage of this work is robustness to different types of heart sounds gained from not requiring segmentation, the system was tested under different noise levels. Some points that have to be made about the training set are as follows. First, these sounds were recorded for instruction purposes and so are likely to be of better quality than actual clinical recordings. However, at this stage in the study, this issue was postponed for the purpose of preliminary evaluation of the proposed method. Second, since the number of normal and abnormal samples were imbalanced, the normal set was expanded by randomly replicating samples such that its size is comparable to the abnormal set (oversampling). This was done so that the classifier will not be biased toward the majority class [25]. Finally, the sounds came from different sources and thus were recorded with different equipment. This is a potential source of bias since the frequency response for each equipment are different. However this bias is potentially limited because there are more than two recording instruments, but only two classes. Thus, judging by the high classification performance, the classifier is not simply detecting the difference in frequency response. Such a scenario would have resulted in a much lower performance. Moreover, features based on envelope signal are insensitive to variations in frequency response, since frequency information has mostly been discarded during the envelope detection process. In conclusion, due to these issues associated with the current training set, we would like to emphasize that this study is an initial evaluation of the proposed method. More experiments using real clinical sounds recorded with actual equipment, that will be used in the final auscultation device, are needed. This will be addressed in further work.

**Table 1:** Heart Sound Types in the Training Set

Heart Sound Type	Number of Files
Normal	12
Third Heart Sound	4
Fourth Heart Sound	3
Ejection Sound	2
Systolic Click	2
Summation Gallop	1
Opening Snap	2
Split S2	4
Aortic Regurgitation (m)	6
Aortic Stenosis (m)	6
Mitral Regurgitation (m)	6
Mitral Stenosis (m)	5
Pulmonary Stenosis (m)	4

This table lists the type of heart sounds that were used in the experiment. The (m) indicate the heart sound is a murmur.

### 3.1 Evaluation Criterion

**Figure 13:**
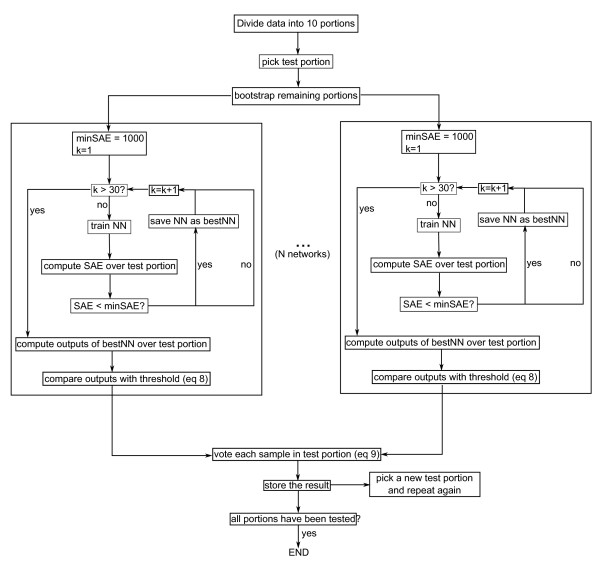
**Cross-validation procedure where *k *is the number of repetitions that each NN is trained in each fold**. SAE is the sum of absolute error defined in Equation 10. The process inside each box is the training for each network.

Ten-fold cross validation that incorporates bagging classifiers was used as performance evaluation; the flowchart of which is shown in Figure 13. It consists of dividing the data into ten portions. Then, in each fold, one portion was reserved for testing while the remaining nine became the training set, which was sampled according to the bootstrapping procedure discussed in section 2.5. This generated *N *different training sets for each of the *N *neural networks. The networks were trained and their performance was evaluated on the test portion, which is the same for all networks since the test portion was not sampled. Each network was trained 30 times and the instances that yielded the best performance were kept. This procedure is illustrated in Figure 13 inside the bounding boxes. Each box represents the training process of a single network. A network's performance was evaluated by the sum of absolute error (SAE), defined in Equation 10:

(10) $$\text{SAE}=\sum_{\forall i\in t}|y_{i}-d_{i}|,$$

where *t *is set of all instances of the test portion, *y_i _*is an actual output of a network and *d_i _*is the desired output (0 for normal and 1 for abnormal).

After the best *N *networks had been obtained, each of them classified the test portion. The outputs that were higher than the threshold *T *were rounded to one, while those that were not were rounded to zero. If the number of ones for a heart sound were more than $\text{floor}(\frac{N}{2})$ where *N *is the number of networks, then that heart sound was classified as abnormal. After all heart sounds in the test portion had been classified, a different portion was selected as test portion and the whole procedure was repeated until all portions had been a test portion.

The reason why the networks had to be trained 30 times was because a network's performance depends on its initial random weights, which determined the starting point on the error function. The error function of a neural network is generally not convex. Thus, the steepest descent may get caught in a local minimum during the training process and hence the need to repeat training many times and keep the best performing network.

Cross-validation results can be quantified by 4 numbers: true positive (TP), false positive (FP), true negative (TN), and false negative (FN),

• TP: the ratio between samples which are actually positive over the number of samples classified as positive.

• FP: the ratio between samples which are actually negative over the number of samples classified as positive.

• TN: the ratio between samples which are actually negative over the number of samples classified as negative.

• FN: the ratio between samples which are actually positive over the number of samples classified as negative.

where positive means diseased heart sounds and negative mean healthy heart sounds. From these raw scores, 3 more indicators of performance can be calculated by:

(11) $$\text{accuracy}=\frac{\text{TP}+TN}{N}$$

(12) $$\text{sensitivity}=\frac{\text{TP}}{\text{TP}+\text{FN}}$$

(13) $$\text{specificity}=\frac{\text{TN}}{\text{TN}+\text{FP}}$$

where *N *is the number of samples in the training set. Finally, a single indicator of classification performance combines both the sensitivity and specificity into a single quantity called the geometric mean [25]. This quantity was used to assess performance in all experiments.

(14) $$g=\sqrt{\text{sensitivity}\times \text{specificity}}$$

### 3.2 Experiment 1A - Determining the Optimal Number of Hidden Neurons

**Figure 14:**
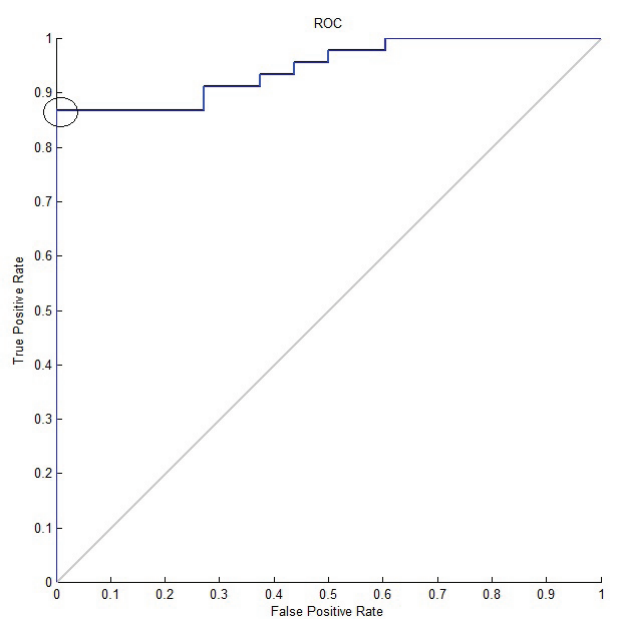
**ROC curve from cross-validation using a single network with 5 hidden neurons**.

The purpose of experiment 1A was to determine the optimal parameters for the classifier which can be used for later experiments. Only a single neural network was used, with decision threshold *T *fixed at 0.5. Its classification performance was tested with increasing number of hidden neurons until there is no increase in performance. This was performed three times and the average was taken. The result is shown in Table 2. There was only a slight performance increase when the number of hidden neurons was increased from 5 to 10. Thus the experiment was stopped and 10 was chosen to be the optimal number of hidden neurons, which was fixed for all later experiments. The receiver operating characteristic (ROC) curve in Figure 14 was plotted to determine the best value for the threshold *T *, where the optimal point on the ROC curve is marked with a circle. The corresponding threshold value was around *T *= 0.15 and this value was used as the decision threshold for all later experiments. Also, from experiment 2A onward, bagging predictors with five networks was used. In summary, the optimal parameters determined by experiment 1A are 10 hidden neurons and *T *= 0.15.

**Table 2:** Result of Experiment 1A

Hidden neurons	trial 1	trial 2	trial	average
1	0.89	0.86	0.89	0.88
5	0.91	0.90	0.92	0.91
10	0.92	0.94	0.91	0.92

Cross validation result using only a single neural network and varying the number of hidden neurons. All performance values in this and all subsequence tables are given in the geometric mean defined in Equation 14.

### 3.3 Experiment 2A - Robustness to White Noise Test

The goal of experiment 2A was to test the robustness of the system against additive noise. Gaussian white noise was added to all heart sounds at signal-to-noise-ratio (SNR) of 15, 10 and 5 dB. At each SNR, cross validation was performed to check the robustness of the system. The result is shown in Table 3. It can be seen that for an SNR of 15 dB and 10 dB, there was little difference from the noise-free case. However, at 5 dB the performance noticeably decreased. This test showed that the system has some robustness to white noise, but in actual operation, loud background noise is something to be careful of.

**Table 3:** Result of Experiment 2A

SNR	trial 1	trial 2	trial 3	average
15 dB	0.92	0.93	0.93	0.93
10 dB	0.88	0.91	0.91	0.90
5 dB	0.86	0.81	0.82	0.83

Cross validation result where the training set had white noise added at different SNR's.

### 3.4 Experiment 2B - Robustness to Impulse Noise Test

In actual operation of the system in a hospital, it may encounter impulse-like noise. It is likely that the patient may move during recording, causing impulse-like noise in the recorded heart sound due to friction between the stethoscope's chest-piece and the patient's chest. In this experiment the robustness of the system against impulse-like noise was tested. A total of 16 sounds of both normal and abnormal type had impulse noise of various duration (0.1, 0.2 and 0.3 s) added by replacing a segment of a signal with random sequence with range {-max max} where max is the maximum value of that signal. Cross-validation was performed with these sounds mixed in the training set. The result is shown in Table 4. Impulse noise of all durations had little affect on the system's performance. In practice, impulse noises are generally less than 0.3 seconds long so the system can be considered robust to impulse noise.

**Table 4:** Result of Experiment 2B

Noise duration	trial 1	trial 2	trial 3	average
0.1 s	0.92	0.92	0.90	0.91
0.2 s	0.90	0.89	0.92	0.90
0.3 s	0.91	0.90	0.89	0.90

Impulse noise robustness test: some heart sounds in the training set had impulse noise of different duration added.

### 3.5 Experiment 2C - Robustness to Unseen Abnormal Heart Sound Types

In this experiment the goal was to assess the robustness of the system to abnormal heart sound types that were not part of the training set. Different abnormal sound types were left out of the training process and were used to test the system trained on the remaining sounds. The result is shown in Table 5. It can be seen that in most cases the left-out sounds have been correctly classified.

**Table 5:** Result of Experiment 2C

Unseen type	Accuracy
AR	4/5
AS	5/6
MR	5/5
MS	4/4
PS	3/3
S3	4/4
S4	3/3
Split S2	3/4
Ejection Sounds	2/2
Systolic Click	2/2
Opening Snap	2/2

Unseen abnormal heart sound types test, each trial a type of abnormal heart sound was left out of the training set. The system was then tested on these unseen sound types.

### 3.6 Discussion and Comparison with Other Works

The goal of this work is to provide a heart sound analysis algorithm for an automatic auscultation device. Such device will be used mostly in rural clinics as an early and cheap detection for heart disease, not as a main diagnosis device. This is the main reason why we decided to restrict the problem to be two-classes, and focus on incorporating as many type of abnormal sounds as possible, and to be robust to noise.

From the experiments, it can be seen that the proposed method achieved average performance of 0.92 (all performance numbers are geometric means unless stated otherwise) for the noise-free case and 0.9 under additive white noise of 10 dB. This provides quite a comfortable noise margin, especially in rural clinics that tend to be noisy. In the extreme case where the noise level was increased to 5 dB, classification performance was significantly decreased to 0.83. A separate examination area, with the auscultation device placed as far away as space permits from any noise sources such as air conditioning, should be able to provide an SNR level within the robust range.

In actual auscultation, a patient may move slightly during recording. Such movement causes impulse noise which may affect the classification result. Experiment 2B showed that impulse noise up to 0.3 second in duration may be tolerated with only slight decrease in accuracy. Based on these results it can be concluded that if the SNR is kept higher than 10 dB and long impulse noise is relatively rare; one could expect around 0.90 classification performance from the proposed method.

The final experiment showed how the proposed method can be generalized to classify abnormal sound types that were not part of the training set. In practice such cases should be rare since the system must be trained extensively before being fielded, but it provides some confidence knowing that the algorithm is likely to generalize correctly if such a case does occur. Even split S2, which is hard to detect, was mostly classified correctly under ideal noise-free conditions.

It is difficult to make direct comparison between this work and others, because the goals are different, and there is no standard test set upon which to compare. Therefore, we discuss some general comments on how the proposed method compares with other works in this field. In terms of classification accuracy alone, this method was not the best. Several studies reported over 98% classification accuracy. This makes our method seem inferior, especially when the focus is only on two-class situations, while many studies are multi-class, and identified the disease that a heart sound is showing. Our method however, has a wide coverage. There were 12 different types of abnormal heart sound in the training set, which is more than most other studies that often include only the four main types of murmur: aortic regurgitation/stenosis and mital regurgitation/stenosis. A diverse training set that includes abnormal sounds that are very similar to normal (split S2, small S3 and S4), makes accurate classification quite difficult even for the two-class case. The training set also included sounds such as that in Figures 3 and 7 that are very difficult to segment automatically, due to very large murmur and/or diminished FHS. In such situations the proposed method is highly advantageous since segmentation is not required, which is the unique point of this algorithm. The only "segmentation" performed is cropping out segments of heart sound signals and their envelopes with lengths equal to 5 cardiac cycles of each respective sound. These segments can begin and end anywhere within a cardiac cycle, that is, the beginning of the segments do not have to align with the beginning of a cardiac cycle. This means that classification accuracy will not be affected by erogenous feature vectors produced by miss-segmented cycles.

Robustness against noise is another main advantage of the proposed method, as indicated by the result of experiment 2A and 2B. Extracting multiple cardiac cycles to form a feature vector averages out the effect of any peculiarity in a particular cycle, such as impulse noise. Focusing only on two classes and skipping segmentation make the proposed method seem overly simple, but it allowed for high robustness and coverage while not compromising practical use. This is because even if a patient has been diagnosed with a particular disease by heart sound analysis and referred to a hospital, then the patient will most likely be re-examined using techniques other than auscultation before the condition is confirmed. Thus the proposed method is suitable for its intended use.

On the negative side, the training set used may have introduced bias, since sounds came from different sources and the normal set was over-sampled. Moreover, most sounds in the training set were recorded for instruction purpose so they are likely to be of better quality than actual clinical recordings. These issues will be addressed in further research by building a new training set using the same equipment, and recorded in the same environment where the final automatic auscultation device will be deployed. The method will be evaluated again using this new training set.

## 4 Conclusions

A new approach to heart sound analysis was proposed that does not require segmentation. The method is applicable to a wide range of heart sounds, from normal to those containing severe murmurs where one of the FHS may disappear. The training set incorporated 12 different types of abnormal heart sounds ranging from split S2 to severe murmurs. Using geometric mean as index of performance and ten-fold cross validation, experiments were conducted to verify the effectiveness of the proposed method and to determine the optimal configuration for the classifier. Based on these parameters, further tests were conducted to assess the robustness of the system to white noise, impulse noise, and unseen heart sound types. The experiments showed that the system can operate at 10 dB SNR, and with 0.3 s long impulse noise with average performance of 0.9. In conclusion, the advantages of this work are:

1. It does not require segmentation.

2. It is applicable to a wide range of heart sounds.

3. It is robust to noise.

While the shortcomings of this work are:

1. It only classifies heart sounds as normal or abnormal.

2. The training set may have introduced bias due to different frequency response and oversampling of the normal set.

### 4.1 Further Work

Further works include stand-alone software implementation and field testing in a hospital using a new training set to concretely validate the proposed method. Also another approach based on single-class classification (SCC) may be explored. In SCC the training data consists of samples from only a single class, which is usually the "normal" class. Applying SCC to heart sound analysis reformulates the problem to be detection of healthy heart sounds and the classifiers would be trained using only healthy samples. This approach eliminates the need to collect abnormal samples and would make data collection much easier.

## Competing interests

The authors declare that they have no competing interests.

## Authors' contributions

KT conceived of the study, procured the equipment and collaboration from Thammasat University hospital, and is the project leader. WK suggested ideas for features, edited the manuscript and provided some samples. AN outlined the envelope detection module and tested the code. SY coded the algorithm, conducted the experiments, and drafted the manuscript. All authors read and approved the final manuscript.
